# Emergence of 16S rRNA Methylase Gene *rmtB* in *Salmonella Enterica Serovar* London and Evolution of RmtB-Producing Plasmid Mediated by IS*26*

**DOI:** 10.3389/fmicb.2020.604278

**Published:** 2021-01-15

**Authors:** Jing Wang, Zhen-Yu Wang, Yan Wang, Fan Sun, Wei Li, Han Wu, Peng-Cheng Shen, Zhi-Ming Pan, Xinan Jiao

**Affiliations:** ^1^Key Laboratory of Prevention and Control of Biological Hazard Factors (Animal Origin) for Agrifood Safety and Quality, Ministry of Agriculture of China, Yangzhou University, Yangzhou, China; ^2^Jiangsu Key Laboratory of Zoonosis/Jiangsu Co-Innovation Center for Prevention and Control of Important Animal Infectious Diseases and Zoonoses, Yangzhou University, Yangzhou, China; ^3^College of Animal Science and Technology, Jilin Agricultural Science and Technology University, Jilin, China

**Keywords:** cointegration, IncN, IS*26*, *rmtB*, *Salmonella*

## Abstract

This study aimed to characterize 16S rRNA methylase genes among *Salmonella* and to elucidate the structure and evolution of *rmtB*-carrying plasmids. One hundred fifty-eight *Salmonella* isolates from one pig slaughterhouse were detected as containing 16S rRNA methylase genes; two (1.27%) *Salmonella* London isolates from slaughtered pigs were identified to carry *rmtB*. They were resistant to gentamicin, amikacin, streptomycin, ampicillin, tetracycline, florfenicol, ciprofloxacin, and sulfamethoxazole/trimethoprim. The complete sequences of RmtB-producing isolates were obtained by PacBio single-molecule real-time sequencing. The isolate HA1-SP5 harbored plasmids pYUHAP5-1 and pYUHAP5-2. pYUHAP5-1 belonged to the IncFIB_K_ plasmid and showed high similarity to multiple IncFIB_K_ plasmids from *Salmonella* London in China. The *rmtB*-carrying plasmid pYUHAP5-2 contained a typical IncN-type backbone; the variable region comprising several resistance genes and an IncX1 plasmid segment was inserted in the resolvase gene *resP* and bounded by IS*26*. The sole plasmid in HA3-IN1 designated as pYUHAP1 was a cointegrate of plasmids from pYUHAP5-1-like and pYUHAP5-2-like, possibly mediated by IS*26* via homologous recombination or conservative transposition. The structure differences between pYUHAP1 and its corresponding part of pYUHAP5-1 and pYUHAP5-2 may result from insertion, deletion, or recombination events mediated by mobile elements (IS*26*, IS*CR1*, and IS*Kpn43*). This is the first report of *rmtB* in *Salmonella* London. IncN plasmids are efficient vectors for *rmtB* distribution and are capable of evolving by reorganization and cointegration. Our results further highlight the important role of mobile elements, particularly IS*26*, in the dissemination of resistance genes and plasmid evolution.

## Introduction

Aminoglycosides such as gentamicin and amikacin have been widely used to treat infections caused by Gram-negative bacteria in clinical settings (human and animal), and they also used for growth promotion in animal husbandry. Resistance to aminoglycosides is due to enzymatic modification/inactivation of aminoglycosides, mutation or modification of aminoglycoside-binding site (16S rRNA of 30S ribosomal subunits), decreased permeability, and augmented efflux (Doi et al., 2016). Among them, 16S rRNA methylases are of great concern for conferring high-level resistance to all aminoglycosides used to treat systemic infections. Since the identification of *armA* in 2003, 10 16S rRNA methylase genes (*armA*, *rmtA*, *rmtB*, *rmtC*, *rmtD*, *rmtE*, *rmtF*, *rmtG*, *rmtH*, and *npmA*) have been identified and globally disseminated in *Enterobacteriaceae* (Galimand et al., 2003; Doi et al., 2016). Although 16S rRNA methylase genes, especially *armA* and *rmtB*, are widely disseminated in *Escherichia coli* isolates from various sources, low prevalence is observed in *Salmonella* (Folster et al., 2009; Doi et al., 2016; Fang et al., 2019). *Salmonella* spp. are one of the leading causes of foodborne illness, and contaminated food, particularly animal-derived food products, are the main sources of *Salmonella* infections for humans (European Food Safety Authority and European Centre for Disease Prevention and Control [EFSA and ECDC], 2018; Marus et al., 2019). The dramatic increase of antibiotic resistance in *Salmonella* has been a global health concern.

Mobile genetic elements play a critically important role in the acquisition and dissemination of resistance genes in Gram-negative and Gram-positive bacteria (Partridge et al., 2018). Cointegration between plasmids via homologous recombination or IS*26*-mediated replicative or conservative transposition has been previously described, allowing plasmids to acquire more resistance or virulence genes (Sun et al., 2016; Wong et al., 2017; Hua et al., 2020).

In this study, we aimed to investigate the prevalence of 16S rRNA methylase genes among *Salmonella* isolates from one pig slaughterhouse in Jiangsu province, China, and to elucidate the structure and evolution of *rmtB*-carrying plasmids.

## Materials and Methods

### Bacterial Strains and 16S rRNA Methylase Genes Detection

One hundred fifty-eight *Salmonella enterica* isolates including *Salmonella* Derby (*n* = 69), *Salmonella* Typhimurium (*n* = 33), *Salmonella* Rissen (*n* = 28), *Salmonella* London (*n* = 16), *Salmonella* Chester (*n* = 5), *Salmonella* Yoruba (*n* = 2), *Salmonella* Indiana (*n* = 2), *Salmonella* Pakistan (*n* = 2), and *Salmonella* Enteritidis (*n* = 1) were previously obtained from pig carcass swab samples, environmental samples, equipment samples, and intestinal content samples in one slaughterhouse in Huai’an, Jiangsu province, 2016 (Zhou et al., 2017). The presence of 16S rRNA methylase genes (*rmtA*, *rmtB*, *rmtC*, *rmtD*, *rmtE*, *rmtF*, *rmtG*, *rmtH*, and *armA*) was detected by PCR and sequencing (Supplementary Table S1).

### Antimicrobial Susceptibility Testing

The *rmtB*-positive isolates were determined for MICs of ampicillin, cefotaxime, meropenem, gentamicin, amikacin, streptomycin, tetracycline, florfenicol, ciprofloxacin, colistin, and sulfamethoxazole/trimethoprim using microbroth dilution method. The results were interpreted according to EUCAST^1^. The *E. coli* strain ATCC 25922 was used for quality control.

### Plasmid Transferability and Stability

Conjugation experiments were performed using streptomycin-resistant *E. coli* C600 as the recipient strain as previously described (Chen et al., 2007). Transconjugants were selected on MacConkey agar plates containing 3,000 mg/L streptomycin and 32 mg/L amikacin (pYUHAP5-2 and pYUHAP1) or chloramphenicol (pYUHAP5-1). Conjugal transfer frequencies were calculated as the number of transconjugants per recipient; experiments were performed in triplicate.

The stability of *rmtB*-carrying plasmids pYUHAP5-2 and pYUHAP1 in the original strains was investigated by passage in daily refreshed (100-fold dilution) antibiotic-free LB broth for 7 days. On the last day, the cultures were streaked on LB agar plates. 100 colonies were replica-plated on LB agar plates with amikacin (32 mg/L) and were randomly selected to confirm the presence of *rmtB* by PCR.

### Whole Genome Sequencing and Analysis

The whole genome of *rmtB*-positive isolates was extracted and sequenced using PacBio single-molecule real-time sequencing (RSII platform) (Pacific Biosciences, Menlo Park, CA). Raw sequence data were introduced into the nonhybrid Hierarchical Genome Assembly Process (HGAP version 4). The plasmid sequences were analyzed and annotated by the RAST server^2^, ResFinder^3^, PlasmidFinder^4^, and BLAST^5^.

### Nucleotide Sequence Accession Number

The whole genome sequences of strains HA3-IN1 and HA1-SP5 have been deposited in the GenBank database under accession number PRJNA648279.

## Results and Discussion

### Identification of *rmtB* and Antimicrobial Susceptibility

Among the 158 *Salmonella* isolates, two ST155 *Salmonella* London isolates from carcass swab samples (Zhou et al., 2017) were positive for *rmtB*. None of the other 16S rRNA methylase genes were detected in this study. *Salmonella* London was rarely reported before, but it has begun to emerge in various sources (patients, pigs, wild birds, meat products, and the environment) and spread worldwide over the past decades (Shipp and Dickson, 2011; Bonardi et al., 2016; Jurado-Tarifa et al., 2016; Trimoulinard et al., 2017; Chen et al., 2019b). To our knowledge, this is the first report of 16S rRNA methylase genes in *Salmonella* London.

As shown in Table 1, two *rmtB*-positive *Salmonella* London strains HA1-SP5 and HA3-IN1 exhibited high-level resistance to amikacin (MIC > 256 mg/L) and also showed resistance to ampicillin, gentamicin, streptomycin, tetracycline, florfenicol, ciprofloxacin, and sulfamethoxazole/trimethoprim, but susceptibility to cefotaxime, meropenem, and colistin.

**TABLE 1:** Characterizations of RmtB-producing *Salmonella* London in this study.

Strain	Genome size (bp)	Resistance gene/mutation (chromosome)	MIC (μg/mL)											Plasmid (size in bp)	Plasmid replicon	Resistance genes (plasmid)
						
			AMP	CTX	MEM	GEN	AMI	STR	TET	FFC	CIP	CL	SXT			
HA1-SP5	4,747,268	*aac(6’)-Iaa parC* T57S	>128	0.03	0.03	>128	>256	>256	>128	>128	32	0.25	64	pYUHAP5-1 (112,916)	IncFIB_*K*_	*bla*_*TEM*__–1b_, *aac(6’)-Ib-cr*, *aac(3)-IId*, *aadA16*, *strAB*, *tet*(A), *catA2*, *floR*, *mph*(A), *arr3*, *sul1*, *sul2*, *dfrA27*
HA3-IN1	4,746,179	*aac(6’)-Iaa parC* T57S	>128	0.03	0.03	>128	>256	>256	>128	>128	8	0.25	64	pYUHAP1 (176,767)	IncN1, IncX1, IncFIB_*K*_	*bla*_*TEM*__–1b_, *aac(6’)-Ib-cr*, *aac(3)-IId*, *aadA16*, *aadA22*, *aph(3’)-IIa*, *rmtB*, *strAB*, *tet*(A), *catA2*, *floR*, *qnrB6*, *mph*(A), *lnu*(F), *arr3*, *sul1*, *sul2*, *dfrA27*

*AMP, ampicillin; CTX, cefotaxime; MEM, meropenem; GEN, gentamicin; AMI, amikacin; STR, streptomycin; TET, tetracycline; FFC, florfenicol; CIP, ciprofloxacin; CL, colistin; SXT, sulfamethoxazole/trimethoprim.*

### Characterization of *rmtB*-Positive *Salmonella* London Strains

To better understand the genetic features of *rmtB*, complete sequences of the two *rmtB*-bearing *Salmonella* London strains were obtained. The isolate HA1-SP5 consisted of a 4,747,268-bp chromosome and two plasmids pYUHAP5-1 and pYUHAP5-2 (Table 1). The isolate HA3-IN1 consisted of a 4,746,179-bp chromosome and a single plasmid pYUHAP1 (Table 1). Both of them carried the aminoglycoside resistance gene *aac(6’)-Iaa* in the chromosome and mutation in *parC* (T57S) which could partly explain the ciprofloxacin resistance (Table 1).

### The *Salmonella* London Strain HA1-SP5 Plasmid pYUHAP5-1

**FIGURE 1:**
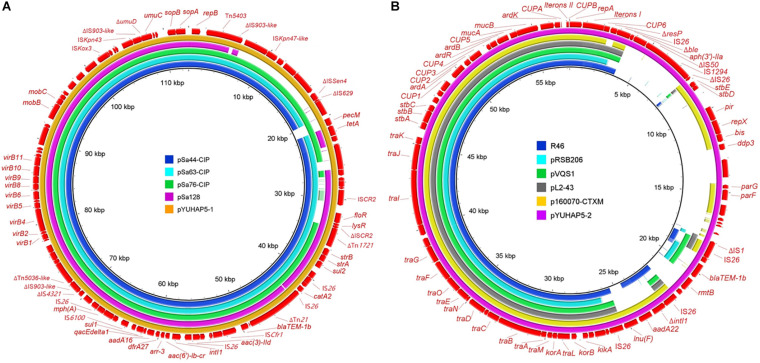
**(A)** Comparison of IncFIB_*K*_ plasmid pYUHAP5-1 in this study with other IncFIB_*K*_ plasmids pSa44-CIP (*Salmonella* London, food, MH430882), pSa63-CIP (*Salmonella* London, meat, MG874043), pSa76-CIP (*Salmonella* London, meat, MG874044), and pSa128 (*Salmonella* London, pork, MG870194) in China. **(B)** Comparison of IncN plasmid pYUHAP5-2 in this study with other IncN plasmids R46 (*Salmonella* Typhimurium, AY046276), pRSB206 (uncultured bacterium, Germany, wastewater treatment plants effluents, JN102344), pVQS1 (*Salmonella* Virchow, Switzerland, human, JQ609357), pL2-43 (*Escherichia coli*, Switzerland, lamb, KJ484641), and p160070-CTXM (*Klebsiella pneumoniae*, China, food, MG288677). The outer circles in red with annotation are the reference plasmids pYUHAP5-1 and pYUHAP5-2, respectively.

The largest plasmid in HA1-SP5, designated as pYUHAP5-1, had a size of 112,916 bp with GC content of 54.93%. It belonged to the IncFIB_*K*_ type and carried 14 resistance genes (Table 1). pYUHAP5-1 was highly similar to plasmids pSa63-CIP (GenBank accession no. MG874043), pSa44-CIP (MH430882), pSa76-CIP (MG874044), and pSa128 (MG870194) carried by *Salmonella* London from meat products in Shenzhen, China (83–98% coverage and 99.9% identity) (Chen et al., 2018; Li et al., 2018; Chen et al., 2019a; Figure 1A). Similar IncFIB_*K*_ plasmids were detected in *Salmonella* isolates, particularly *Salmonella* London (Chen et al., 2018). In line with previous studies (Chen et al., 2018; Chen et al., 2019a), pYUHAP5-1 was conjugative at a high frequency of 4.6 × 10^–1^ transconjugants per recipient.

**FIGURE 2:**
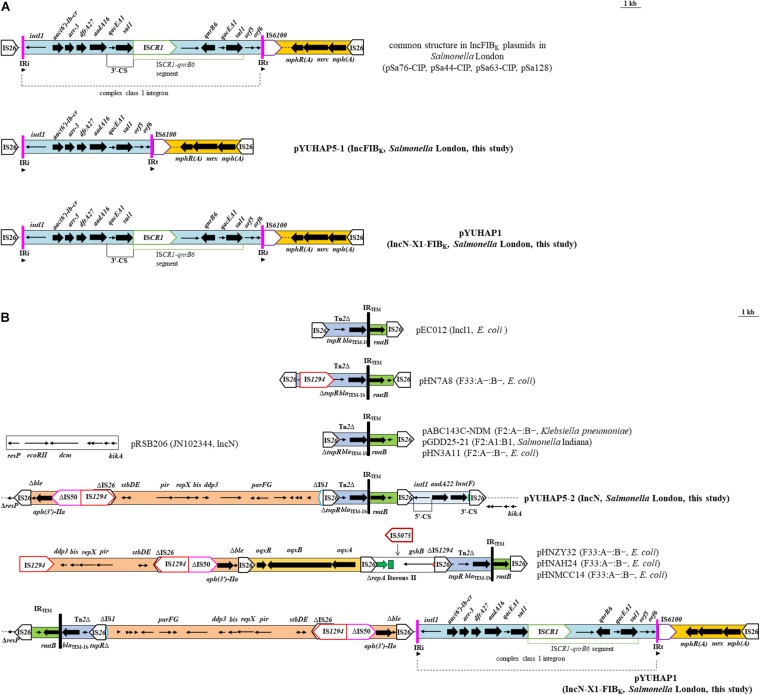
Genetic organization of the multiresistance region of **(A)** IncFIB_*K*_ plasmids pYUHAP5-1 and pYUHAP1. Plasmid pSa128 harbors four copies of IS*CR1-qnrB6* segment. Arrowheads show the relative directions of IR. The IRt was not included in the complex class 1 integron, it was the short remnants of the IRt end of *tni*_402_ flanking IS*6100*. **(B)** IncN plasmids pYUHAP5-2 and pYUHAP1 and structural comparison with plasmids pRSB206 (JN102344), pEC012 (KT282968), pHN7A8 (JN232517), pABC143-NDM (MH316136), pHN3A11 (JX997935), pHNZY32 (MG197502), pHNAH24 (MG197495), and pHNMCC14 (MG197498). The extents and directions of antibiotic resistance (thick arrows) and other genes are indicated. Δ indicates a truncated gene or mobile element. ISs are shown as boxes labeled with their name. Tall bars represent the inverted repeats (IR) of transposon.

The primary feature of these IncFIB_*K*_ plasmids is the co-location of *qnrB6-aac(6′)-Ib-cr* within a complex class 1 integron (*intI1-aac(6′)-Ib-cr-arr-3-dfrA27-aadA16-qacE*Δ1-*sul1*-IS*CR1-qnrB6-qacE*Δ1-*sul1*). It was followed by the *mph*(A) region [*mph*(A)-*mrx-mphR*(A)] flanked by IRt-IS*6100* and IS*26* (Figure 2A). The unique difference between these IncFIB_*K*_ plasmids and pYUHAP5-1 was the absence of ∼5.5 kb IS*CR1-qnrB6* segment in pYUHAP5-1 (Figure 2A). IS*CR1* can mobilize itself and adjacent DNA segments, including resistance genes, into the 3*’*-conserved segment (3*’*-CS) by rolling circle transposition (Toleman et al., 2006). The common IncFIB_*K*_ plasmids in *Salmonella* London (e.g., pSa63-CIP) may evolve from pYUHAP5-1-like plasmids by acquiring *qnrB6* through IS*CR1*-mediated transposition within class 1 integron. A circular intermediate containing IS*CR1*, adjacent region, and part of the 3′-CS is created, and it can be rescued via homologous recombination with the 3′-CS of class 1 integron, resulting in duplications of this circular intermediate (Toleman et al., 2006). The IS*CR1-qnrB6* segment in tandem repeats has been previously described in IncFIB_*K*_ plasmids (Li et al., 2018).

### The *Salmonella* London Strain HA1-SP5 Plasmid pYUHAP5-2

The *rmtB*-carrying plasmid pYUHAP5-2 was 57,187-bp with a GC content of 49.76%, and belonged to IncN plasmid group. IncN plasmids have been described as vectors for *rmtB* dissemination in *E. coli*, but F2:A1:B1 plasmids were mainly associated with *rmtB* spread in *Salmonella* (Yao et al., 2011; Fang et al., 2019). pYUHAP5-2 contained the typical IncN backbone encoding functions for replication, conjugal transfer, maintenance, and stability, and showed high similarity (>99% identity) to other IncN plasmids such as the archetypal IncN1 plasmid R46 (AY046276) (Figure 1B). As observed in other IncN plasmids (e.g., R46, pRSB206, pVQS1), repetitive elements (CUP1-6, CUPA, CUPB, A1, and A2), which function as regulatory elements (Delver and Belogurov, 1997), and iteron regions I and II (with tandem repeats of 37 bp) for plasmid replication and copy number control (Krishnan and Iyer, 1990; Papp and Iyer, 1995) were also identified on plasmid pYUHAP5-2 (Figure 1B), but differed by the numbers of 37-bp repeats within iteron region I (Supplementary Table S2). pYUHAP5-2 was stably maintained within the original isolate for 7 days without selection, and could be transferred to *E. coli* C600 at a frequency of 1.9 × 10^–4^ transconjugants/recipient.

The variable region (∼23.5 kb) of pYUHAP5-2 was inserted in the resolvase gene *resP* and bounded at both ends by IS*26*, resulting in the deletion of 3,513-bp segment (Δ*resP-ecoRII-dcm*) compared to pRSB206 (JN102344, IncN1) (Figure 2B). The variable region consisted of four IS*26* elements flanking three different parts. The first part (∼14.6 kb) contained resistance genes Δ*ble* (bleomycin resistance) and *aph(3’)-IIa* (aminoglycoside resistance); mobile elements ΔIS*50*, IS*1294*, and ΔIS*26*; and IncX1 plasmid region (*stbD*/*E-pir-repX-bis-ddp3-parG*/*F*) truncated by ΔIS*1* (Figure 2B). This part was identical to those of plasmids pACN001-A (KC853434, *E. coli*), pCTXM-2271 (MF589339, *E. coli*), and pCFSA1007-2 (CP033386, *Salmonella enterica*), differed by two to six nucleotide changes. The IncX1 region in pYUHAP5-2 was 99.7% identical to the corresponding part of pOLA52 (EU370913, IncX1) with 89% coverage, whereas *repX* was absent in pOLA52. The 10,044-bp segment (IS*26*-Δ*ble-aph(3’)-IIa-*ΔIS*50*-IS*1294*-ΔIS*26-stbD*/*E-pir-repX-bis-ddp3-*314 bp) was previously detected with opposite orientation in multiple F33:A-:B- plasmids from *E. coli* of various origins in China, such as pHNZY32 (MG197502, patient), pHNAH24 (MG197495, chicken), and pHNMCC14 (MG197498, chicken meat) (Wang et al., 2018; Figure 2B). It highlights the ability of the IncX1 plasmid segment, along with resistance genes, to be captured by distinct plasmids through mobile elements. It is common to observe multiple replication regions within a single plasmid, which may increase its host range (Partridge et al., 2018).

The second part (2,828-bp) consisted of an incomplete Tn*2* (containing β-lactam resistance gene *bla*_*TEM*__–1b_) and *rmtB* and was found in numerous plasmids such as pABC143C-NDM (KY130431, F2:A-:B-) and pGDD25-21 (MH316136, F2:A1:B1) (Figure 2B). This segment was identical to those of F2:A-:B- plasmids or related plasmids, whereas slight difference was observed in other types of plasmids, e.g., pHN7A8 (JN232517, F33:A-:B-) and pEC012 (KT282968, IncI1) (Figure 2B). The last part (2,780-bp) was an incomplete class 1 integron with Δ*intI1* and *aadA22-lnu*(F) cassette flanked by IS*26*, as observed in other plasmids with the same IS*26*/5’-CS and 3’-CS/IS*26* boundary, such as p160070-CTXM (MG288677, *K. pneumoniae*) and pNDM33-1 (MN915011, *E. coli*). Our results further confirm that IS*26* elements may play a vital role in the dissemination of resistance genes and formation of variable region of pYUHAP5-2.

### pYUHAP1 Was Generated by Plasmid Fusion Between pYUHAP5-1-Like and pYUHAP5-2-Like Plasmids

**FIGURE 3:**
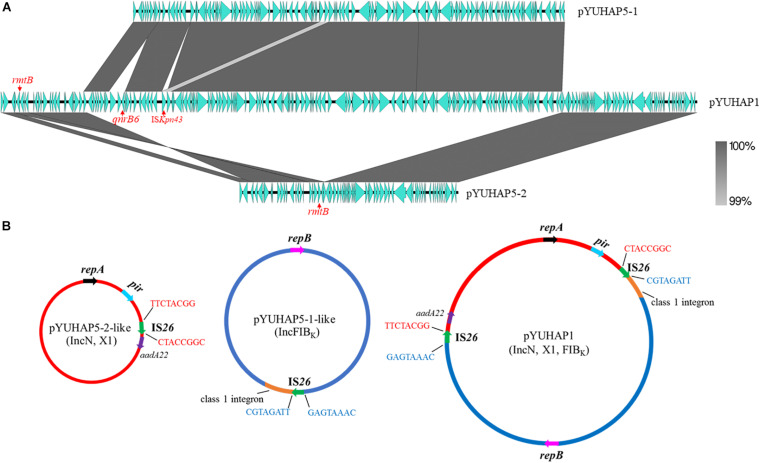
Proposed mechanism for the formation of cointegrate plasmid pYUHAP1. **(A)** Linear sequence comparison of pYUHAP1, pYUHAP5-1, pYUHAP5-2. Green arrows represent ORFs. Regions of homology are shaded in gray. **(B)** Proposed model for the plasmid cointegration by IS*26*. IS*26* involved in the cointegration are shown as green arrows, the 8-bp sequences adjacent to each IS*26* are indicated by black lines with red or blue letters. Colored arrows with black, red, and blue represent replicon genes of IncN, IncFIB_*K*_, and IncX plasmids. Orange segments represent the complex class 1 integron shown in Figure 2A.

The sole plasmid in HA3-IN1, namely pYUHAP1, was 176,767 bp with an average G+C content of 53.14%. It harbored three replicons, including IncN1, IncX1, and IncFIB_*K*_ (Table 1). pYUHAP1 was a cointegrate plasmid comprised of sequence from pYUHAP5-1-like and pYUHAP5-2-like plasmids (Figure 3). The IncN1-X1 RmtB-producing plasmid region of pYUHAP1 was similar to pYUHAP5-2, except that the first and second parts of variable region in pYUHAP5-2 (Δ*ble-aph(3’)-IIa-*ΔIS*50*-IS*1294*-ΔIS*26-stbD*/*E-pir-repX-bis-ddp3-*ΔIS*1*-IS*26*-Tn*2-rmtB*) was in the opposite orientation with a shorter 157-bp IS*1* in pYUHAP1 (Figure 2B). This could be readily explained by homologous recombination between IS*26* located in inverse orientations. Additionally, the pYUHAP5-1-derived region in pYUHAP1 was 119,756 bp, the size difference was due to the acquisition of 5,466-bp IS*CR1-qnrB6* segment within the class 1 integron, and the presence of one copy of IS*Kpn43* (1,374 bp) located between ΔIS*4321* and IS*903*-like in pYUHAP1 (Figure 3A). The presence of additional plasmid-mediated quinolone resistance gene *qnrB6* could easily explain the difference of MIC of ciprofloxacin between HA1-SP5 and HA3-IN1 (Table 1). The regions of pYUHAP1 derived from the different ancestor plasmids were separated by a pair of IS*26*, the cointegrate formation was likely generated by IS*26* (Figure 3B). We did not observe additional copies of IS*26* or 8-bp target-site duplication, suggesting that this cointegration may occur by IS*26*-mediate homologous recombination or conservative transposition rather than replicative transposition (He et al., 2015; Harmer and Hall, 2016). The insertion, deletion, or recombination events mediated by mobile elements (e.g. IS*26*, IS*CR1*, IS*Kpn43*) have occurred to form plasmids as ancestors for pYUHAP1 or occurred after cointegration, but the precise series of events cannot be determined with the available data.

Similar cointegration events mediated by IS*26* have occurred between IncN1-F33:A-:B- plasmids and *mcr-1*-carrying phage-like plasmid in *E. coli* (He et al., 2019), IncN, F33:A-: B-, and rolling-circle plasmids in *Proteus mirabilis* (Hua et al., 2020), or virulence plasmid and IncHI2 resistance plasmid in *Salmonella* Enteritidis (Wong et al., 2017). IS*26* elements play a vital role in the dissemination of resistance genes, formation of mosaic resistance regions, and reorganization of plasmids in Gram-negative bacteria (He et al., 2015; Partridge et al., 2018). They also indicate that cointegration formation between plasmids is an important but not rare way for plasmids to evolve and capture more resistance genes, virulence genes, or other beneficial genes for their dissemination and maintenance. In this study, pYUHAP1 acquired more resistance genes through cointegration between two resistance plasmids, but it remained stable (100%) and conjugative with frequency of 1.03 × 10^–4^ transconjugants/recipient.

## Conclusion

This study revealed the emergence of *rmtB* in *Salmonella* London of swine origin in China. The fusion of *rmtB*-carrying IncN plasmid and IncFIB_*K*_ multiresistance plasmid was mediated by IS*26*. IncN plasmids have become efficient vectors for *rmtB* transmission not only in *E. coli*, but also in *Salmonella*, and are able to evolve by reorganization and co-integration. Our results further confirm the critical role of mobile elements, particularly IS*26*, in the mobilization of resistance genes, formation of resistance regions, and diversity of plasmid structures.

## Data Availability Statement

The datasets presented in this study can be found in online repositories. The names of the repository/repositories and accession number(s) can be found in the article/Supplementary Material.

## Author Contributions

Z-MP, XJ, and JW conceived the study. YW, Z-YW, FS, HW, P-CS, and JW carried out the experiments. JW, Z-YW, and WL analyzed the data. JW wrote the manuscript. Z-MP and XJ revised the manuscript. All authors read and approved the final manuscript.

## Conflict of Interest

The authors declare that the research was conducted in the absence of any commercial or financial relationships that could be construed as a potential conflict of interest.

## Footnotes

1 https://mic.eucast.org/Eucast2

2 https://rast.nmpdr.org/rast.cgi

3 https://cge.cbs.dtu.dk//services/ResFinder

4 https://cge.cbs.dtu.dk/services/PlasmidFinder/

5 http://blast.ncbi.nlm.nih.gov/Blast.cgi
