# Supplementary material for: Emergence of 16S rRNA Methylase Gene rmtB in Salmonella Enterica Serovar London and Evolution of RmtB-Producing Plasmid Mediated by IS26
Source: Front Microbiol. 2021 Jan 15;11:604278. doi: 10.3389/fmicb.2020.604278 (PMC7843705; doi:10.3389/fmicb.2020.604278)
Supplement: Supplementary file 1 [file Data_Sheet_1.docx]

**Supplementary data**

**Table S1.** Primers used for PCR and DNA sequencing in this study

| **Gene** | **Primer name** | **Sequence (5’ to 3’)** | **Size (bp)** | **Reference** |
| --- | --- | --- | --- | --- |
| *rmtA* | rmtA-F | AGCTTTGACGATGCCCTAGC | 716 | Chen et al., 2007 |
|  | rmtA-R | CCAATGGTCTTGGTATCCTC |  |  |
| *rmtB* | rmtB-F | ACATCAACGATGCCCTCAC | 725 | Chen et al., 2007 |
|  | rmtB-R | AAGTTCTGTTCCGATGGTC |  |  |
| *rmtC* | rmtC-F | GCCAAAGTACTCACAAGTGG | 741 | Chen et al., 2007 |
|  | rmtC-R | ACCCAACAAGATCATTCTCG |  |  |
| *rmtD* | rmtD-F | CGG CAC GCG ATT GGG AAG C | 635 | Doi et al., 2007 |
|  | rmtD-R | CGG AAA CGA TGC GAC GAT |  |  |
| *rmtE* | rmtE-F | GATGCCGTGTCTGTTACGCCG | 446 | Corrêa et al., 2014 |
|  | rmtE-R | ACGTGAACCCACGAGTCCTGC |  |  |
| *rmtF* | rmtF-F | CGATCCTACTGGGCTCCAT | 314 | Corrêa et al., 2014 |
|  | rmtF-R | GGCATAGTGCTTTTCCATGC |  |  |
| *rmtG* | rmtG-F | ACGGAATGCCGCGCGAAGTA | 381 | Corrêa et al., 2014 |
|  | rmtG-R | TCTCCGCAAGCAGATCGCCG |  |  |
| *rmtH* | rmtH-F | ATGACCATTGAACAGGCAGC | 464 | Corrêa et al., 2014 |
|  | rmtH-R | AGGGCAAAGGTAAAATCCCA |  |  |
| *armA* | armA-F | CAATCAGGGGCAGTTATCA | 529 | Chen et al., 2007 |
|  | armA-R | CCCTATAACCTTCGAATC |  |  |

**References**

Chen, L., Chen, Z.L., Liu, J.H., Zeng, Z.L., Ma, J.Y., and Jiang, H.X. (2007). Emergence of RmtB methylase-producing *Escherichia coli* and *Enterobacter cloacae* isolates from pigs in China. *Journal of Antimicrobial Chemotherapy* 59**,** 880-885. doi: 10.1093/jac/dkm065.

Corrêa, L. L., Montezzi, L. F., Bonelli, R. R., Moreira, B. M., and Picão, R. C. (2014). Revised and updated multiplex PCR targeting acquired 16S rRNA methyltransferases. *International Journal of Antimicrobial Agents* 43, 479–481. doi:10.1016/j.ijantimicag.2014.02.003

Doi, Y., and Arakawa, Y. (2007). 16S ribosomal RNA methylation: emerging resistance mechanism against aminoglycosides. *Clin Infect Dis* 45, 88-94. doi: 10.1086/518605.

**Table S2. Characteristics of IncN plasmids analyzed in this study**

| **Plasmid** | **Size (bp)** | **Host** | **Resistance genes** | **Replicon type** | **37-bp repeat in Iterons I** | **GenBank** |
| --- | --- | --- | --- | --- | --- | --- |
| pYUHAP5-2 | 57,187 | *Salmonella* London | *bla*_TEM-1b_, *aadA22*, *aph(3’)-IIa*, *rmtB*, *lnu*(F) | IncN1-IncX1 | 32 | PRJNA648279 |
| pYUHAP1 | 176,767 | *Salmonella* London | *bla*_TEM-1b_, *aac(6’)-Ib-cr*, *aac(3)-IId*, *aadA16*, *aadA22*, *aph(3’)-IIa*, *rmtB*, *strAB*, *tet*(A), *catA2*, *floR*, *qnrB6*, *mph*(A), *lnu*(F), *arr3*, *sul1*, *sul2*, *dfrA27* | IncN1-IncX1-IncFIB_K_ | 32 | PRJNA648279 |
| R46 | 50,969 | *Salmonella* Typhimurium | *bla*_OXA-2_, *aadA1*, *tet*(C), *sul1* | IncN1 | 5 | AY046276 |
| pRSB206 | 52,809 | Uncultured | *bla*_TEM-1b_, *strAB*, *tet*(A), *sul2*, *dfrA14* | IncN1 | 36 | JN102344 |
| p160070-CTXM | 66,643 | *Klebsiella pneumoniae* | *bla*_CTX-M-65_, *aadA22*, *lnu*(F), *fosA3* | IncN1 | 38 | MG288677 |
| pVQS1 | 40,995 | *Salmonella* Virchow | *bla*_TEM-1b_, *qnrS1* | IncN1 | 3 | JQ609357 |
| pL2-43 | 43,265 | *Escherichia coli* | *bla*_CTX-M-1_, *mph*(A) | IncN1 | 6 | KJ484641 |
